# Oleic acid improves pathological changes in Aβ1–42-induced astrocytes and Alzheimer’s disease mouse models through PKA/ACACB/CPT1A

**DOI:** 10.3389/fnins.2026.1771310

**Published:** 2026-04-15

**Authors:** YiBo Xie, Jianan Tian, Hui Li, Yahui Peng, Mingjie Li, Yun Wu

**Affiliations:** 1 Department of Neurology, Second Affiliated Hospital of Harbin Medical University, Harbin, Heilongjiang, China; 2 Department of Biochemistry and Molecular Biology, School of Basic Medicine, Harbin Medical University, Harbin, Heilongjiang, China; 3 Harbin Medical University, Harbin, Heilongjiang, China

**Keywords:** ACACB, Alzheimer’s disease, astrocyte, fatty acid, oleic acid

## Abstract

**Background:**

Alzheimer’s disease (AD) is the most prevalent neurodegenerative disorder. Emerging evidence indicates that fatty acid oxidation is impaired in both patients with AD and AD animal models. In the brain, fatty acid metabolism occurs predominantly in astrocytes. Diets enriched in monounsaturated fatty acids (MUFAs) are often recommended for individuals with AD. Oleic acid (OA), a common dietary MUFA, has been shown to reduce amyloid plaque accumulation in transgenic mouse models of AD. Moreover, OA decreases the expression of acetyl-CoA carboxylase beta (ACACB/ACC2), a key regulator of fatty acid β-oxidation. However, the precise mechanism by which OA may alleviate amyloid plaque deposition through modulation of brain fatty acid metabolism remains unclear.

**Objective:**

To determine whether dietary OA supplementation partially restores astrocytic fatty acid metabolism by suppressing ACACB and enhancing fatty acid β-oxidation, thereby attenuating AD-related pathology.

**Methods:**

Using NHANES 2011–2014 data, we applied survey-weighted multivariable logistic regression to examine the association between energy-adjusted MUFA intake and low cognitive function, with adjustment for multiple testing. We then screened GEO datasets to identify AD-associated genes involved in fatty acid metabolic dysregulation, identifying ACACB as a candidate target. The expression of ACACB and its downstream effector carnitine palmitoyltransferase 1A (CPT1A) were validated in mouse and cell models. APP/PS1 mice received dietary OA supplementation, followed by behavioral testing and brain histopathological analyses. In parallel, an Aβ1–42-induced astrocyte injury model was used to assess lipid droplet accumulation, mitochondrial function, and cellular energy metabolism. ACACB knockdown/overexpression and CPT1A overexpression were used to test pathway-specific effects of OA.

**Results:**

Higher MUFA intake was associated with better cognitive function. ACACB as a key fatty acid metabolism-related gene in AD. In APP/PS1 mice, OA improved cognitive performance and reduced Aβ plaque deposition, accompanied by decreased ACACB and increased CPT1A expression in brain tissue. *In vitro*, OA modulated ACC2 activity through protein kinase A (PKA) signaling, increased fatty acid β-oxidation, reduced lipid droplet accumulation, restored mitochondrial membrane potential and ATP production, and enhanced astrocyte-mediated support of neuronal synaptic growth.

**Conclusion:**

OA ameliorates AD-related pathology and cognitive impairment by restoring astrocytic fatty acid β-oxidation through the PKA/ACACB/CPT1A pathway.

## 1 Instruction

Alzheimer’s disease (AD), the most common type of dementia in older adults (Twarowski and Herbet, 2023), accounts for 60 to 70% of all dementia cases (Silva et al., 2019). Approximately 50 million people worldwide are currently living with AD, a number expected to rise to 82 million by 2030 and 152 million by 2050. Between 2000 and 2019, the reported AD-related death count rose by over 145% (Alzheimer’s Association Report, 2023; Alzheimer’s Association Report, 2024). Early detection and prevention hold potential for mitigating this escalating public health concern.

The risk of AD is estimated to be influenced by genetic factors to an extent of 60–80% (Scheltens et al., 2021). Neuronal loss, synaptic degeneration, and the formation of amyloid peptide aggregates known as senile plaques are the pathological changes of AD (Azam et al., 2021). Early intervention can delay the onset of pathological features (Morris et al., 2015). Some studies suggest that a healthy lifestyle can help delay disease progression. Evidence indicates that physical exercise can preserve cognitive function and attenuate cognitive decline in older adults, including individuals with normal cognition (del Pozo et al., 2022), mild cognitive impairment (MCI) (de Oliveira Silva et al., 2019), and AD (Balbim et al., 2022). Multiple studies have emphasized the positive effects of a healthy diet on disease (Stefaniak et al., 2022; Xu et al., 2021; van den Brink et al., 2019), emphasizing the Mediterranean diet and the Dietary Approaches to Stop Hypertension diet (Amtul et al., 2011), both of which advocate the intake of unsaturated fatty acids.

Recent studies have demonstrated that lipid homeostasis is disrupted in the early stages of AD (Yin, 2023), with pathological changes such as lipid droplet (LD) deposition documented. Notably, LD levels are reportedly higher in glial cells (Farmer et al., 2019). Lipids are fundamental components for cellular function, serving critical roles in membrane formation, intercellular signaling, energy storage, and homeostasis maintenance. In the brain, lipid dysregulation is associated with the etiology and progression of neurodegenerative diseases and other neurological pathologies (Yoon et al., 2019). Fatty acids (FAs) also play a crucial role in the growth and development of neurons (Farmer et al., 2019). Free FAs in the extracellular space can enter cells either directly by diffusion or via specific FA transporters. Once inside the mitochondrion, fatty acids are first converted to fatty acyl-CoA, which is then converted to acylcarnitine by CPT1A to allow it to traverse the inner mitochondrial membrane (Knottnerus et al., 2018). Upon entering the mitochondrial matrix, these long-chain acyl-CoAs (LC-acyl-CoAs) undergo repeated cleavage via β-oxidation in a cyclical enzymatic process, yielding acetyl-CoA until the FA is fully oxidized. The resulting acetyl-CoA molecules then enter the tricarboxylic acid (TCA) cycle and ultimately generate ATP through mitochondrial oxidative phosphorylation (Kemp et al., 2024).

FAs cross the blood–brain barrier (BBB) by binding with fatty acid transporters (Zhang et al., 2024). After entering the brain, they are taken up by neural cells and can be stored as LDs (Zhang et al., 2024). Both neurons and astrocytes express multiple FA transporters, including fatty acid transport protein 1 (FATP1), fatty acid transport protein 4 (FATP4), and cluster of differentiation 36 (CD36) (Barber and Raben, 2019). Within the brain, fatty acid β-oxidation occurs predominantly in astrocytes (Qi et al., 2021). In addition to taking up circulating free fatty acids (FFAs), astrocytes also acquire FAs released from neighboring cells. When FAs accumulate excessively in neurons, neurons secrete apolipoprotein E (ApoE)-containing lipoprotein particles, which are subsequently internalized by astrocytes (Ioannou et al., 2019). Astrocytes then direct these fatty acids to mitochondria for β-oxidation and oxidative phosphorylation (Barber and Raben, 2019).

Clinical studies have shown that serum levels of acetyl-L-carnitine and other acylcarnitines decline progressively from normal cognition to MCI and, ultimately, AD. Because acylcarnitines are key intermediates in fatty acid catabolism, these findings suggest that fatty acid catabolism is impaired in patients with AD (Cristofano et al., 2016; Ciavardelli et al., 2016). Studies have found that FA levels in the mitochondria of astrocytes from 4-month-old 5xFAD mice are significantly higher than those in age-matched wild-type (WT) mice, confirming defects in mitochondrial fatty acid degradation in astrocytes (Mi et al., 2023). Abnormal oxidative phosphorylation in astrocytes induces LD accumulation, resulting in synaptic loss, neuroinflammation, demyelination, and cognitive decline (Mi et al., 2023). Morant-Ferrando et al. (2023) demonstrated that knockout of CPT1A in adult mouse astrocytes resulted in cognitive impairment. Similarly, Ju et al. (2024) found that reduced CPT1A expression in astrocytes led to lipid dyshomeostasis, thereby promoting astrocytic damage and neuroinflammation, which ultimately contributed to the pathogenesis of AD. Furthermore, astrocytes are involved in the clearance of amyloid-beta (Aβ). In this respect, studies have found that in the entorhinal cortex of APP/PS1 mice and AD patients, glial fibrillary acidic protein (GFAP)-positive mouse and human astrocytes are densely clustered around β-amyloid plaques (Chen et al., 2022; Preman et al., 2021).

Study has demonstrated that a diet high in oleic acid (OA, an unsaturated fatty acid) can decrease amyloid plaque accumulation in the brains of AD transgenic mouse models (Kandel et al., 2022). OA can trigger the development of hippocampal neurons (Natali et al., 2007) and inhibit acetyl-CoA carboxylase (ACC) (Natali et al., 2007). ACC exists in two isoforms, they are ACC1 and ACC2. ACC2 produces malonyl-CoA, which regulates fatty acid β-oxidation in mitochondria by inhibiting CPT1 (Pang et al., 2021). In brain tissue, upon neuronal activation, the release of norepinephrine (NA) and vasoactive intestinal peptide (VIP) activates adenylyl cyclase receptors. As cyclic AMP (cAMP) concentrations increase, cAMP-dependent protein kinase (PKA) phosphorylates ACC, thereby reducing its activity. This leads to decreased malonyl-CoA synthesis and reduced inhibition of CPT1, thereby enabling CPT1 to transport fatty acids for β-oxidation (Yin, 2023).

Based on this background, we hypothesize that incorporating OA into the diet could delay the progression of cognitive impairment. To validate this hypothesis, we conducted an experiment in which mice were divided into three groups: (1) littermate control mice on a standard diet; (2) APP/PS1 mice on a standard diet; and (3) APP/PS1 mice on a diet supplemented with OA. To investigate the specific mechanism of action of OA in patients, data was retrieved from the Gene Expression Omnibus (GEO) online database. We found that ACACB was a key gene associated with fatty acid metabolism in AD. We hypothesized that OA may enhance cognition by modulating the expression of this key gene. No studies have hitherto systematically examined whether OA can improve cognition through fatty acid metabolism. Our findings aims to confirm the hypothesis that OA enhances cognition by downregulating ACACB expression and regulating fatty acid β-oxidation in astrocytes.

## 2 Materials and methods

### 2.1 Database

Diet-related data were sourced from the National Health and Nutrition Examination Survey (NHANES) database^1^ (CDC, 2025). The data have been anonymized. According to the NHANES analytic guidelines, data from the 2011–2012 and 2013–2014 cycles were combined. To account for the complex sampling design, the stratification variable (SDMVSTRA), primary sampling unit variable (SDMVPSU), and Day 1 dietary sample weight (WTDRD1) were incorporated into all analyses. Dietary intake was derived from 24-h dietary recall data and energy-adjusted using the density method, expressed as g/100 kcal. Between-group comparisons were performed using survey-weighted *t*-tests or Wilcoxon rank-sum tests for continuous variables and Rao–Scott *χ*^2^ tests for categorical variables. Survey-weighted multivariable logistic regression models were fitted to evaluate the associations between dietary factors and low cognitive performance, and odds ratios (ORs) with 95% confidence intervals (CIs) were reported. *p*-values were adjusted using the Benjamini–Hochberg false discovery rate (FDR) method, and a two-sided FDR-adjusted *p* < 0.05 was considered statistically significant.

Transcriptome data from datasets GSE109887 and GSE122063 were obtained from the GEO database.^2^ Both datasets are derived from humans and have been anonymized. Genes associated with fatty acid metabolism were obtained from the Molecular Signatures Database (MsigDB)^3^ (Liberzon et al., 2011). The examination of gene expression in different cell types was completed using the AlzData website^4^ (Zhang et al., 2019; Xu et al., 2018). We screened for key genes associated with diseases and fatty acid metabolism. Using the “limma” package in R, differentially expressed genes (DEGs) were identified based on the criteria of |log2 fold change (FC)| >1 and *p*-value <0.05. We used “pheatmap” and “ggplot2” to generate volcano plots and heat maps, screened genes related to fatty acids, and performed least absolute shrinkage and selection operator (LASSO) analysis to obtain key genes. We employed the STRING website^5^ and Cytoscape 3.9.1 software to analyze protein–protein interactions and construct a protein interaction network. The obtained network was examined using the MCODE plugin in Cytoscape to identify key module genes. Finally, we screened for genes intersecting the two sets for subsequent functional studies.

### 2.2 Animals

Kunming (KM) pups were obtained from the Animal Experiment Center of Harbin Medical University. APP/PS1 mice and wild-type littermate controls were purchased from Jiangsu Wukong Biotechnology Co., Ltd., and genotyping was performed. The male-to-female ratio was 1:1. Four-month-old mice were maintained at 25 ± 1 °C under a 12:12-h light–dark cycle. This study complied with the standards of the National Institutes of Health Guide for the Care and Use of Laboratory Animals (NIH Publication No. 80-23, revised 1978).

### 2.3 Diet

APP/PS1 mice and littermate mice were allocated into three groups (1): littermate mice on a standard diet (*n* = 10) (2), APP/PS1 mice on a standard diet (*n* = 10), and (3) APP/PS1 mice on a diet supplemented with OA (*n* = 10). The mice were maintained on the respective diets for 8 weeks, with daily monitoring of food intake, water consumption, and overall health. The conventional diet included the following ingredients: corn, soybean meal, fish meal, wheat flour, yeast powder, vegetable oil, salt, and a mixture of vitamins and minerals (moisture content ≤10%, crude protein ≥18%, crude fat ≥4%, crude fiber ≤5%, crude ash ≤8%, calcium 1.0–1.8%, total phosphorus 0.6–1.2%). The OA-supplemented diet consisted of the conventional diet supplemented with 2% oleic acid (oleic acid purchased from RHAWN, diet purchased from Beijing Keao Xieli Feed Co., Ltd.). Each cage housed five mice, and food intake was recorded per cage. The recorded values were then used to estimate the caloric load per mouse (see Supplementary Table 1).

### 2.4 Y-maze test

The Y-maze consisted of three symmetrical arms (35 cm × 5 cm × 10 cm), with each arm forming a 120° angle with the others. Test lighting conditions were adjusted to 30 ± 5 lux to reduce anxiety in mice. Before testing, all arms were wiped with 75% alcohol. Testing proceeded only after the alcohol had completely evaporated. Tested and untested mice were housed in separate cages. Spontaneous alternation test: Mice were given 5 min to explore the maze freely. The number of times each mouse entered each arm and the order in which they entered different arms were recorded. The alternation ratio was calculated using the formula: [(spontaneous alternation count)/(total arm entries − 2) × 100%]. Novel arm test: A partition blocked one arm of the maze, and the mice were allowed to explore the maze freely for 10 min. During the testing phase, the partition was removed, and the mice were permitted to explore the maze freely for 5 min. The time spent and distance traveled in the novel arm were recorded. Food reward test: The mice were trained to reach the specific arm of the maze where food rewards were placed. During the test phase, the time taken for the mice to enter the specific arm was recorded.

### 2.5 Tissue

At the end of the 8-week feeding period, mice were euthanized by intraperitoneal injection of tribromoethanol. Brain tissue was removed immediately. A portion was rapidly frozen in liquid nitrogen and stored at −80 °C (*n* = 4). The hippocampus was isolated for Western blotting and PCR. The remaining brain tissue (*n* = 6) was fixed in 4% paraformaldehyde for paraffin embedding and histological analyses.

### 2.6 Immunohistochemistry, immunofluorescence, and HE staining

Paraffin-embedded sections of mouse brain tissue were prepared. Brain regions were identified according to a mouse stereotaxic brain atlas (Paxinos and Franklin), and coronal sections containing the hippocampus (CA1, CA3, and dentate gyrus) and the prefrontal cortex were collected at levels between −1.5 mm and −2.5 mm from bregma. Immunohistochemical staining was performed using primary antibodies against ACACB (1:100 dilution, Cat. No. A67611, Nature Bioscience) and MAP2 (1:200 dilution, Cat. No. A22205, Abclonal). Horseradish peroxidase–conjugated goat anti-rabbit IgG (1:50 dilution, Cat. No. A0208, Beyotime Biotechnology) and a goat anti-rabbit fluorescent antibody (1:50 dilution, Cat. No. A0562, Beyotime Biotechnology) were used as secondary antibodies. For immunohistochemistry, DAB staining was carried out using a DAB substrate kit (Beyotime Biotechnology, China), and color development was allowed to proceed for 1 min at room temperature before the sections were observed under a light microscope (Nikon, Japan). After incubation with the fluorescent secondary antibody, nuclei were counterstained with DAPI (Beyotime Biotechnology, China) for 5 min and then examined under a fluorescence microscope (Olympus, Japan). Hematoxylin and eosin (H&E) staining was performed using a commercial staining kit (G1120, Solarbio).

### 2.7 Western blot analysis (WB)

Protein expression levels in brain and cell samples were assessed by Western blotting. Protein concentrations were determined using the BCA method (SW101-02, SevenBio). Samples were mixed with loading buffer (LT101S, Epizyme, Shanghai, China) and subjected to electrophoresis on a 10% Bis-Tris gradient gel (PG112, Epizyme, Shanghai, China) at 80/120 V. Proteins were transferred to PVDF membranes at 400 mA. Membranes were blocked with rapid blocking buffer (P0269, Beyotime Biotechnology) for 15 min and incubated overnight at 4 °C with primary antibodies: ACACB (1:1000, A67611, Nature Biosciences), CPT1A (1:5000, 15184-1-AP, Proteintech), tubulin (1:5000, gift from Dr. Wang Jia), β-actin (1:20000, 81115-1-RR, Proteintech), PSD95 (1:5000, 20665-1-AP, Proteintech), SYN (1:20000, 17785-1-AP, Proteintech), p-ACC2 (1:1000, AP1410PM, ABclonal), and p-PKA (1:1000, AP0557, ABclonal). After washing, membranes were incubated with horseradish peroxidase-conjugated goat anti-rabbit IgG (1:1000, A0208, Beyotime Biotechnology) for 1 h at room temperature. Signals were visualized using a chemiluminescence imaging system (GelView 6000Plus, Guangzhou Biolight Biotechnology Co., Ltd., China).

### 2.8 RT-qPCR

RNA was extracted from cell and tissue samples using TRIzol. Following extraction, RNA concentration was measured using a UV spectrophotometer (Shimadzu, Japan). Reverse transcription and PCR were conducted using a commercial kit (Code No. 639505, Takara Bio; SYBR qRT-PCR Master Mix, Roche). The primers used were:

**Table:** 

	Forward (5′ ⟶ 3′)	Reverse (5′ ⟶ 3′)
β-actin	AGGGAAATCGTGCGTGACAT	TCCAGGGAGGAAGAGGATGC
ACACB	AGAAGCGAGCACTGCAAGGTTG	GGAAGATGGACTCCACCTGGTT
CPT1A	GGCATAAACGCAGAGCATTCCTG	CAGTGTCCATCCTCTGAGTAGC
GFAP	TAACGACTATCGCCGCCAAC	CATTTGCCGCTCTAGGGACT
IL-1β	TCATACACCGACGCCAACAA	TTCTTGAGACCACCGAAGGC
IL-6	TACCACTTCACAAGTCGGAGGC	CTGCAAGTGCATCATCGTTGTTC
TNF	GGTGCCTATGTCTCAGCCTCTT	GCCATAGAACTGATGAGAGGGAG
MAP2	GCTGTAGCAGTCCTGAAAGGTG	CTTCCTCCACTGTGGCTGTTTG
SYN	TGGCTTCGTGAAGGTGCTGCA	ACTCTCCGTCTTGTTGGCACAC
PSD95	TCAGACGGTCACGATCATCGCT	GTTGCTTCGCAGAGATGCAGTC

### 2.9 Cell experiments

#### 2.9.1 Cell culture and in vitro model construction

C8-D1A astrocytes (Cat. No. CL-0506, Procell) were purchased from Procell and cultured in the corresponding complete medium (CM-0506, Procell). The medium was replaced every 2–3 days, and all experiments were performed according to cell growth status. We compared ACACB and CPT1A protein expression between primary astrocytes and the C8-D1A cell line and found no significant difference (Supplementary Figure 2). Considering the experimental limitations (including primary-cell handling and throughput), we used the cell line for subsequent mechanistic experiments. To establish the *in vitro* injury model, astrocytes were treated with Aβ1–42 (GL Biochem, Shanghai, China) at concentrations ranging from 1 to 100 μM for 24, 48, and 72 h. Cell viability was measured using a CCK-8 assay kit (C0037, Beyotime Biotechnology). Based on CCK-8 results, the Aβ1–42 concentration and incubation time that reduced cell viability by approximately 50% were selected as the treatment conditions for subsequent experiments. To establish the OA-treated cell model, cells were exposed to OA at concentrations of 10–50 μM, and the concentration showing no significant effect on cell viability was selected for subsequent experiments. OA was warmed in a 37 °C water bath, dissolved in DMSO, and then diluted into culture medium to the indicated final concentrations.

#### 2.9.2 Astrocyte–neuron co-culture

Primary cell isolation and culture were performed as follows. Cerebral cortex tissue was collected from neonatal mice (0–24 h), and the meninges and blood vessels were removed. The tissue was minced and digested with 2% papain (G8430, Solarbio) at 37 °C for 30 min to facilitate cell dissociation. The cell suspension was then centrifuged and filtered through a 70-μm cell strainer to obtain primary neurons. Six-well plates were pre-coated with poly-L-lysine (PB180522, Procell) at 37 °C for 16 h and washed with PBS before seeding. Primary neurons were seeded and maintained at 37 °C in 5% CO₂. The medium was replaced after 4 h, and culture was continued. After 24 h, pre-adhered and treated astrocytes cultured in Transwell inserts were transferred into the six-well plates. Astrocytes and primary neurons were co-cultured for 1 week to assess neuronal synaptic growth.

### 2.10 Cell transfection

After astrocytes were treated under different conditions, transient transfection was performed according to the manufacturer’s instructions (GenePharma) using a commercial transfection reagent. Forty-eight hours after transfection with overexpression plasmids and siRNAs (both from GenePharma), gene overexpression and knockdown models were established (ACACB knockdown, ACACB overexpression, and CPT1A overexpression).

### 2.11 Mitochondrial membrane potential (TMRE) detection

Mitochondrial membrane potential was measured using a commercial kit (C2001S, Beyotime Biotechnology). After cell adhesion, culture medium was removed and 1 mL TMRE working solution (1:1000) was added to each well. Cells were incubated at 37 °C in 5% CO₂ for 30 min. Supernatants were removed, and cells were washed twice with pre-warmed culture medium. Then, 2 mL pre-warmed medium was added, and fluorescence images were acquired immediately.

### 2.12 Lipid droplet detection

Lipid droplet staining was performed using a commercial kit (C2054M, Beyotime Biotechnology). After cell adhesion, medium was removed and cells were fixed with 4% paraformaldehyde for 10 min at room temperature. Cells were washed with PBS, incubated with 1 mL staining solution at 37 °C in the dark for 60 min, washed twice with PBS, and imaged under a fluorescence microscope (Olympus, Japan).

### 2.13 ATP detection

ATP levels were measured using a commercial kit (S0026, Beyotime Biotechnology). All procedures were performed on ice. Culture medium was removed, and 200 μL lysis buffer (for a 6-well plate) was added to each well for 2 min. Samples were centrifuged at 12,000 × g at 4 °C for 5 min, and supernatants were collected. Then, 100 μL ATP detection working solution (1:9) was added per well and incubated at room temperature for 5 min. Next, 20 μL sample or standard was added, mixed, and measured using a microplate reader (Multimode Plate Reader EnVision^®^ 2015, PerkinElmer).

### 2.14 Statistical analysis

Statistical analysis was conducted using GraphPad Prism 10.2.3 (GraphPad Software, La Jolla, CA, United States). Statistical significance between two groups was assessed using Student’s *t*-test. A two-sided *p* < 0.05 was considered statistically significant. (^*^p < 0.05, ^**^p < 0.01, ^***^p < 0.001, and ^****^p < 0.0001).

## 3 Results

### 3.1 Monounsaturated fatty acid consumption and cognitive scores (database analysis)

**Figure 1:**
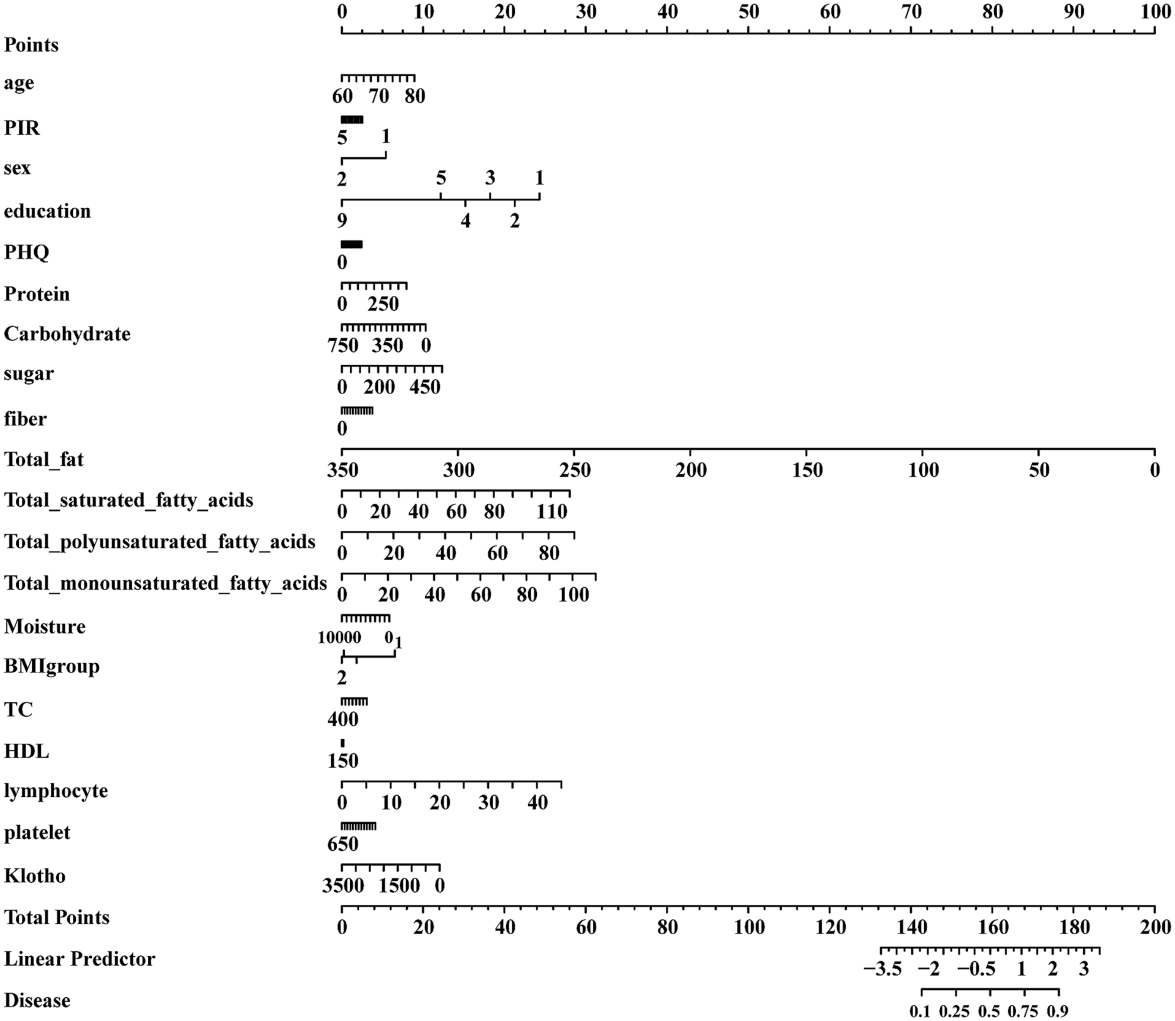
Logistic regression plot of the association between dietary and cognition. The association between various dietary components and low cognitive performance.

Participants exhibiting a CERAD z-score within the lowest quartile were classified as having low cognitive function, while those with missing data were excluded. Ultimately, 962 participants were included in the analysis, comprising 251 with low cognitive function and 711 with normal cognitive function. To analyze the effects of various dietary components on cognitive scores, the following variables were included: age, poverty-income ratio (PIR), sex, Patient Health Questionnaire (PHQ), protein, carbohydrate, sugar, fiber, total fat, cholesterol, total saturated fatty acids (SFAs), total monounsaturated fatty acids (MUFAs), total polyunsaturated fatty acids (PUFAs), BMI, energy, and klotho (a protein that is closely associated with aging, cognitive function). The characteristics of the participants are summarized in Table 1. Older participants (*p* < 0.001), those from low-income families (*p* < 0.001), and those with high PHQ scores (*p* < 0.001) were significantly associated with a greater likelihood of low cognitive function. Specifically, individuals with lower consumption of MUFAs (*p* < 0.05) were more likely to experience cognitive decline. To further investigate the effects of different fatty acid diets on cognitive function, we constructed a logistic regression model based on the corrected dietary data and presented the results using a box-and-whisker plot, as shown in Figure 1. MUFAs were inversely associated with low cognitive function (OR = 0.9899, 95% CI: 0.9899–0.9900, FDR-adjusted *p* = 0.0086, *p* = 0.0047), as shown in Table 2. PUFAs were inversely associated with low cognitive performance (*p* = 0.0606), whereas, after adjustment, total saturated fatty acids were positively associated with low cognitive performance. Fatty acids are classified into saturated and unsaturated categories, with unsaturated fatty acids further divided into MUFA and PUFA varieties. In this study, we observed divergent associations with cognitive function. SFA intake was significantly associated with an increased likelihood of low cognitive scores. In contrast, higher MUFA intake was significantly associated with a lower likelihood of low cognitive performance. No significant association was detected between PUFA intake and cognitive outcomes. Based on these findings, increasing MUFA intake was chosen for subsequent experimental investigation. Previous study has demonstrated that OA can improve amyloid-beta aggregation in both AD cellular models and mouse models (Amtul et al., 2011); however, the mechanisms remain to be elucidated. Therefore, a diet supplemented with OA was selected for subsequent experiments.

**Table 1:** Baseline characteristics of study participants.

Cognitive decline			
Parameter	Normal (*n* = 711)	Low (*n* = 251)	*p*
Age	67.11 (5.32)	69.42 (5.75)	<0.001
PIR	2.84 (1.60)	2.13 (1.49)	<0.001
Sex			<0.001
Male	313 (44.0)	148 (59.0)	
Female	398 (56.0)	103 (41.0)	
PHQ	2.98 (4.08)	4.34 (5.48)	<0.001
Protein	74.28 (34.61)	71.64 (37.39)	0.311
Carbohydrate	221.71 (97.55)	219.67 (104.18)	0.78
Sugar	92.50 (58.06)	92.43 (63.18)	0.988
Fiber	16.95 (10.24)	16.67 (10.75)	0.713
Total fat	73.61 (40.62)	65.54 (37.91)	0.006
Cholesterol	277.03 (209.27)	260.01 (207.96)	0.268
Total saturated fatty acids	23.50 (14.57)	20.30 (12.64)	0.002
Total monounsaturated fatty acids	26.42 (15.46)	23.79 (14.75)	0.019
Total polyunsaturated fatty acids	17.27 (11.21)	15.88 (10.63)	0.087
BMI group			0.062
<18.5 kg/m^2^	6 (0.8)	4 (1.6)	
≥18.5 to <25.0 kg/m^2^	165 (23.2)	67 (26.7)	
25.0 to <30.0 kg/m^2^	253 (35.6)	76 (30.3)	
≥30.0 kg/m^2^	284 (39.9)	99 (39.4)	
Energy	1868.57 (776.34)	1769.92 (815.40)	0.088
Klotho	867.93 (286.92)	811.01 (325.73)	0.009

Data are presented as unweighted numbers (weighted percentages) for categorical variables and mean (SE) for continuous variables. The *p*-value reflects the statistical significance of the correlation between the parameter and low cognition (*p* < 0.05 is considered significant). (Normal: normal cognitive; low: low cognitive).

**Table 2:** Associations between dietary factors and low cognitive function.

Term	OR (95% CI)	*p*-value	FDR-adjusted *p*
Total polyunsaturated fatty acids	0.9985 (0.9972, 0.9999)	0.0386	0.0606
Total fat	1.0001 (0.9998, 1.0003)	0.7107	0.7107
Energy	0.9968 (0.9911, 1.0026)	0.2759	0.3589
Cholesterol	1.0044 (1.0021, 1.0067)	2.34 × 10^−4^	8.59 × 10^−4^
Total monounsaturated fatty acids	0.9899 (0.9899, 0.9900)	0.0047	0.0086
Total saturated fatty acids	1.0032 (1.0014, 1.0050)	4.13 × 10^−4^	9.09 × 10^−4^
Moisture	0.9998 (0.9995, 1.0001)	0.2937	0.3589
Carbohydrate	1.0536 (1.0263, 1.0816)	9.83 × 10^−4^	8.59 × 10^−4^
sugar	1.0097 (0.9820, 1.0382)	0.4953	0.5449
Protein	1.0515 (1.0239, 1.0798)	2.14 × 10^−4^	8.59 × 10^−4^
Fiber	0.9561 (0.9326, 0.9802)	4.10 × 10^−4^	9.09 × 10^−4^

OR >1 indicates increased odds of the outcome, whereas OR <1 indicates decreased odds. Raw *p*-values are reported from the regression model, and FDR-adjusted *p*-values were calculated using the Benjamini–Hochberg procedure to account for multiple testing. Statistical significance was defined as two-sided *p* < 0.05; for multiple comparisons, FDR-adjusted *p* < 0.05 was considered significant.

### 3.2 Oleic acid supplementation mitigates cognitive decline and attenuates neuropathology in APP/PS1 mice

**Figure 2:**
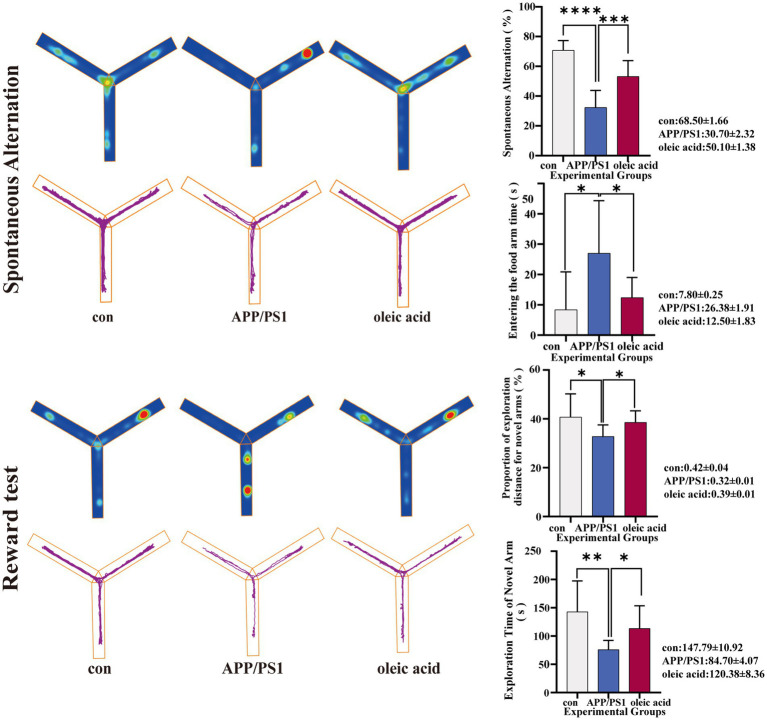
Y maze results (*n* = 10). The percentage of spontaneous alternation is shown for littermate control mice (Con), APP/PS1 mice fed a standard diet (APP/PS1), and APP/PS1 mice fed an oleic acid (OA) diet (APP/PS1 + OA). The results of reward test and novel arm exploration test are also shown in the figure. (Spontaneous alternation: Con vs. APP/PS1 *p* < 0.0001APP/PS1 vs. OA *p* = 0.0005; reward test Con vs. APP/PS1 *p* = 0.0131, APP/PS1 vs. OA *p* = 0.0231; proportion of exploration distance for novel arms (%) Con vs. APP/PS1 *p* = 0.0292, APP/PS1 vs. OA *p* = 0.0133; exploration time of novel arm (s) Con vs. APP/PS1 *p* = 0.0016, APP/PS1 vs. OA *p* = 0.0135). Data are presented as mean ± SEM.

After 8 weeks of dietary intervention, the Y-maze test was conducted, as shown in Figure 2. At 6 months of age, APP/PS1 mice displayed memory impairment, with a significantly reduced spontaneous alternation rate compared to normal mice (*p* < 0.0001) and a prolonged duration spent in the same area. APP/PS1 mice on the OA-supplemented diet also exhibited a reduced spontaneous alternation rate compared to normal mice; however, their performance showed significant improvement compared to APP/PS1 mice on the conventional diet (*p* = 0.0005). In the novel arm test, APP/PS1 mice on the OA-supplemented diet explored the novel arm for a longer duration (*p* = 0.0135) and covered a larger proportion of the exploration area (*p* = 0.0133) than APP/PS1 mice on the conventional diet. Furthermore, in the reward test, the OA-supplemented group located the reward arm more quickly (*p* = 0.0231). Collectively, these results indicate that the OA-supplemented diet enhanced spatial learning and working memory in APP/PS1 mice.

**Figure 3:**
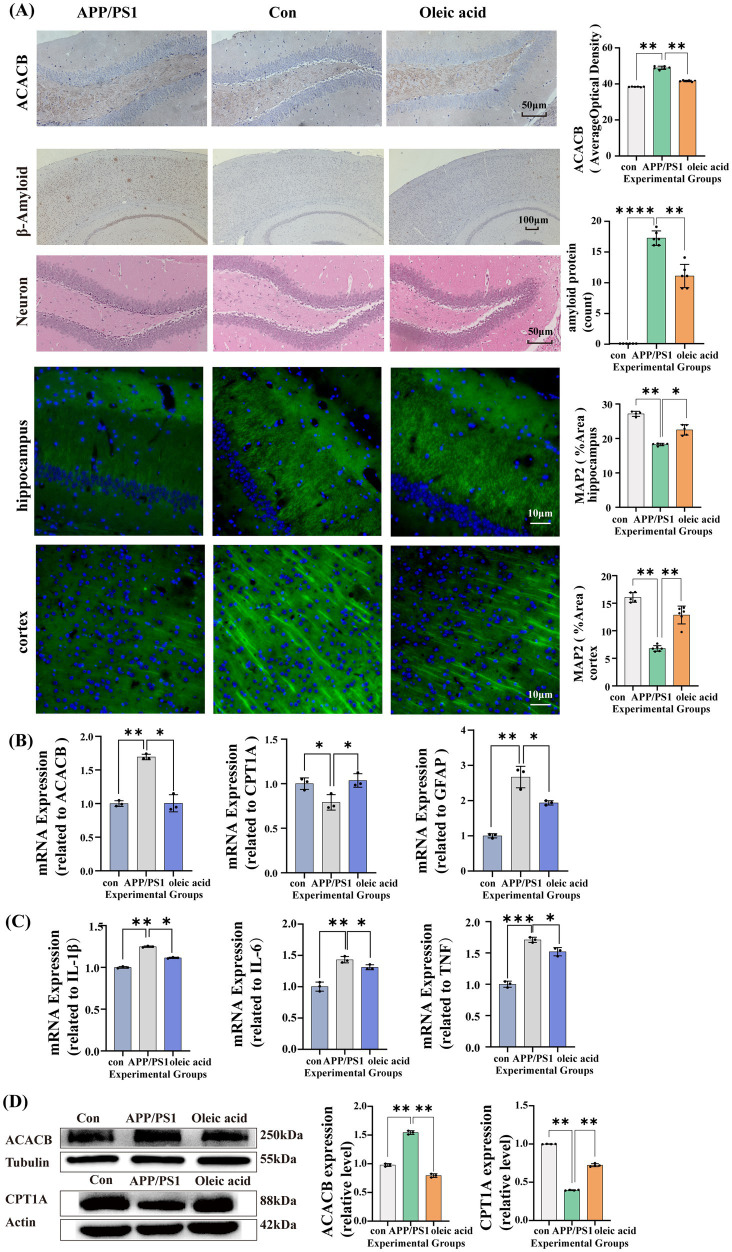
Results of the animal experiments. **(A)** In APP/PS1 mice fed the standard diet (*n* = 6), ACACB immunohistochemical staining was significantly increased compared with littermate controls (*p* = 0.0055), whereas the oleic acid (OA) diet reduced ACACB expression (*p* = 0.0089) (Con: 38.39 ± 0.06; APP/PS1: 45.59 ± 2.04; oleic acid: 38.96 ± 0.15.). APP/PS1 mice on the standard diet also showed a marked increase in β-amyloid plaque burden (*p* < 0.0001), which was attenuated by OA feeding (*p* = 0.0057) (*n* = 6). Hematoxylin and eosin (H&E) staining revealed nuclear atrophy in APP/PS1 mice on the standard diet, and this change was alleviated by the OA diet (*n* = 6). MAP2 immunofluorescence in the hippocampus (*n* = 4) showed lower MAP2 expression in APP/PS1 mice than in controls (*p* = 0.009), while OA-fed APP/PS1 mice showed higher MAP2 expression than standard diet-fed APP/PS1 mice (*p* = 0.03). A similar trend was observed in the cortex (*n* = 6). **(B)** PCR results in mouse brain tissue (*n* = 3) (ACACB: Con vs. APP/PS1 *p* = 0.009, APP/PS1 vs. OA *p* = 0.01; CPT1A: Con vs. APP/PS1 *p* = 0.04, APP/PS1 vs. OA *p* = 0.04; GFAP: Con vs. APP/PS1 *p* = 0.001, APP/PS1 vs. OA *p* = 0.04). **(C)** RT-qPCR results for inflammatory factors in mouse brain tissue (*n* = 3) (IL-1β: Con vs. APP/PS1 *p* = 0.001, APP/PS1 vs. OA *p* = 0.01; IL-6: Con vs. APP/PS1 *p* = 0.001, APP/PS1 vs. OA *p* = 0.01; TNF: Con vs. APP/PS1 *p* = 0.0004, APP/PS1 vs. OA *p* = 0.01). **(D)** Protein expression levels of ACACB and CPT1A in mouse brain tissue (*n* = 3) (ACACB: Con vs. APP/PS1 *p* = 0.0051, APP/PS1 vs. OA *p* = 0.001; CPT1A: Con vs. APP/PS1 *p* = 0.0066, APP/PS1 vs. OA *p* = 0.001). Data are presented as mean ± SEM.

Next, the pathological features in APP/PS1 mice fed an OA-supplemented diet were attenuated compared to those in APP/PS1 mice on a conventional diet. As depicted in Figure 3A, the number of amyloid plaques in the brain tissue of APP/PS1 mice fed the OA-supplemented diet was significantly lower than that in APP/PS1 mice on the conventional diet (*p* = 0.0057). Additionally, at 6 months of age, APP/PS1 mice on the conventional diet already exhibited pathological changes such as nuclear shrinkage. In contrast, APP/PS1 mice fed the OA-supplemented diet had not yet developed these pathological features.

### 3.3 Identification of key genes associated with fatty acid metabolism using online databases

**Figure 4:**
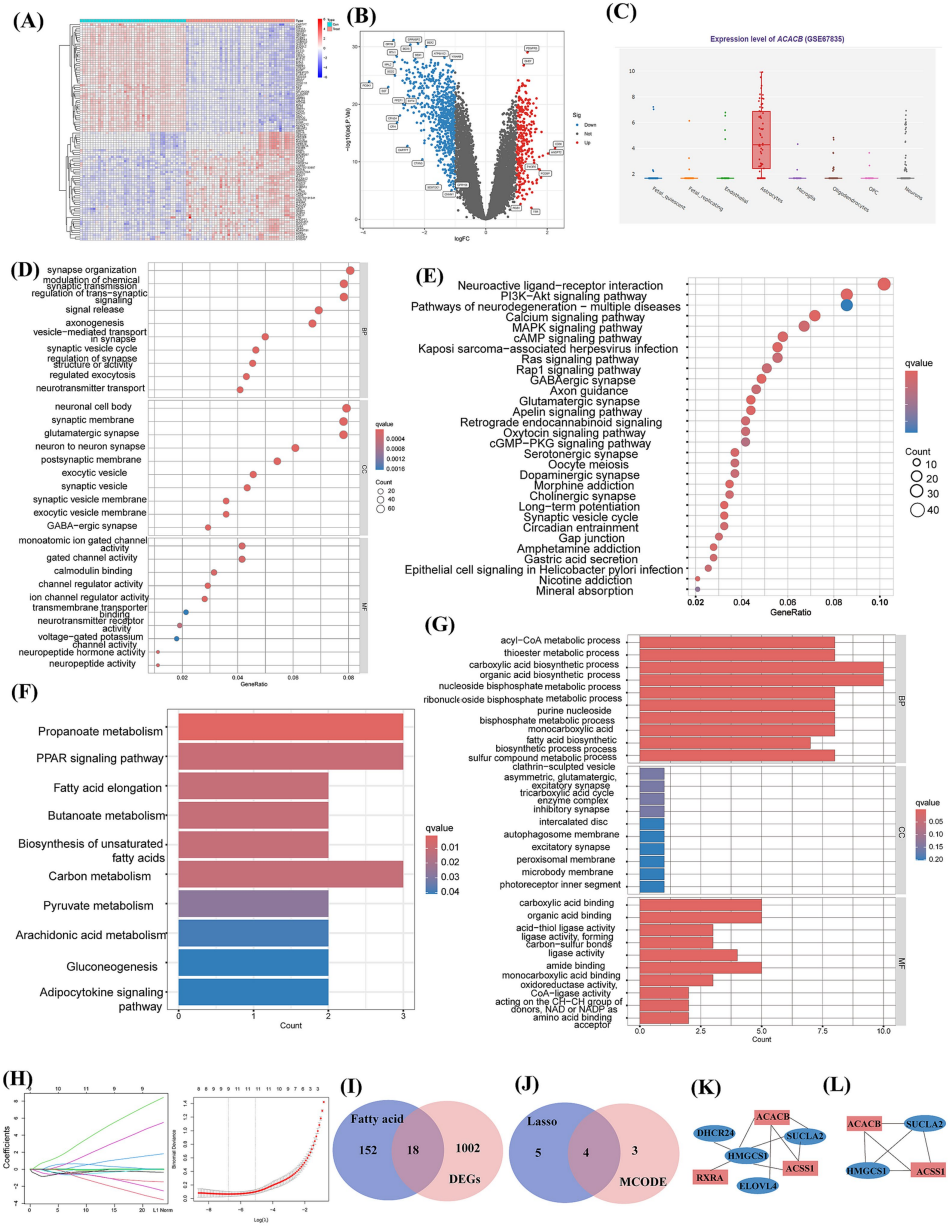
Bioinformatics analysis workflow and key findings. **(A)** Heatmap of differentially expressed genes (DEGs). **(B)** Volcano plot of DEGs. **(C)** Cell-type expression distribution of ACACB. **(D,E)** GO and KEGG enrichment analyses of DEGs. **(F,G)** GO and KEGG enrichment analyses of key fatty acid metabolism-related genes. **(H)** LASSO regression analysis of genes overlapping between Alzheimer’s disease (AD)-related genes and fatty acid metabolism-related genes. **(I)** Venn diagram showing overlap between DEGs and fatty acid metabolism-related genes. **(J)** Overlapping genes identified by LASSO and MCODE analyses. **(K)** Key modules identified by MCODE analysis (blue, downregulated genes; red, upregulated genes). **(L)** Specific overlapping genes from LASSO and MCODE analyses (blue, downregulated genes; red, upregulated genes).

Patients with AD may exhibit dysregulated fatty acid metabolism. To further investigate whether dietary OA can enhance cognitive function by improving intracranial fatty acid metabolism, we utilized the GEO database to screen for key genes associated with fatty acid metabolism in AD patients. As shown in Figure 4, we used the “limma” package in R to screen for differentially expressed genes (DEGs) based on the criteria |log2 fold change (FC)| >1 and *p*-value <0.05. A total of 1,020 differentially expressed genes were identified (306 up-regulated and 714 down-regulated).

Enrichment analyses were performed on DEGs. As shown in Figure 4D, the Gene Ontology (GO) analysis was primarily enriched in terms of synapse organization, modulation of chemical synaptic transmission, regulation of trans-synaptic signaling, synaptic membranes, glutamatergic synapses, neuronal cell bodies, and channel regulator activity, indicating involvement in functions and pathways related to synapse formation and transmission. Figure 4E presents the Kyoto Encyclopedia of Genes and Genomes (KEGG) enrichment analysis. DEGs were primarily enriched in synapse-related pathways. The development of neuronal axons and dendrites is closely linked to astrocytes, and brain fatty acid metabolism is also predominantly regulated by astrocytes. To further explore the association between fatty acid metabolism and AD, we intersected the DEGs with genes related to fatty acid metabolism, yielding 18 intersecting genes (DHCR24, MID1IP1, RXRA, ACOT7, YWHAH, ENO2, CIDEA, GAD2, GABARAPL1, ELOVL4, ACSS1, CYP2C8, ACACB, SLC27A2, HMGCS1, TDO2, AKR1C3 and SUCLA2), as shown in Figure 4I. Protein–protein interaction (PPI) analysis was performed on these 18 genes to further screen for critically essential genes, We utilized MCODE analysis to identify genes within the most critical module, yielding 7 genes: *ACACB*, *ACSS1*, *RXRA*, *DHCR24*, *HMGCS1*, *ELOVL4*, and *SUCLA2*, as shown in Figure 4K. Next, we conducted LASSO analysis on these 18 genes associated with fatty acids, as shown in Figure 4H, identifying nine key genes: YWHAH, SUCLA2, MID1IP1, HMGCS1, GABARAPL1, CIDEA, AKR1C3, ACSS1, and ACACB. By taking the intersection of the two sets, we found four common genes, as depicted in Figure 4L: ACACB, ACSS1, SUCLA2, and HMGCS1. ACACB and ACSS1 were up-regulated genes, while SUCLA2 and HMGCS1 were down-regulated.

### 3.4 OA regulated fatty acid metabolism by downregulating ACACB expression, thereby ameliorating cognitive decline-related pathology in APP/PS1 mice

To further substantiate that OA regulated fatty acid metabolism in the brain tissue of APP/PS1 mice by downregulating ACACB, we extracted brain tissue from the three groups of mice. We confirmed via immunohistochemistry that ACACB expression in APP/PS1 mice on a standard diet was higher than that in APP/PS1 mice on an OA-supplemented diet (*p* = 0.0055), as shown in Figure 3A. To further validate this, we extracted brain tissue from the three groups of mice and conducted PCR and WB experiments. As shown in Figure 3B, ACACB mRNA expression was significantly higher in the brain tissue of APP/PS1 mice (*p* = 0.009), while its downstream carnitine palmitoyltransferase-1A (CPT1A) expression was reduced (*p* = 0.04). In APP/PS1 mice fed an OA-supplemented diet, ACACB mRNA expression decreased compared to those on a standard diet (*p* = 0.01), while CPT1A expression increased (*p* = 0.04). Additionally, GFAP (*p* = 0.001), IL-1β (*p* = 0.001), IL-6 (*p* = 0.001), and TNF (*p* = 0.0004) mRNA expressions in the brain tissues of APP/PS1 mice were significantly higher than in control mice, as shown in Figures 3B,C. Furthermore, GFAP (*p* = 0.04), IL-1β (*p* = 0.01), IL-6 (*p* = 0.01), and TNF (*p* = 0.01) mRNA expressions were lower in APP/PS1 mice fed an OA-supplemented diet, as shown in Figures 3B,C. We further validated the protein expression levels of ACACB and CPT1A in brain tissue. In the APP/PS1 mouse group fed a conventional diet, ACACB expression was significantly higher than that in the control group (*p* = 0.0051), CPT1A expression was significantly lower (*p* = 0.0066). In APP/PS1 mice fed an OA-supplemented diet, ACACB expression was reduced (*p* = 0.001) and CPT1A expression was increased (*p* = 0.001) compared to the conventional diet group, as shown in Figure 3D.

We next validated the expression of ACACB primarily in astrocytes using the online database AlzData, as shown in Figure 4C. Astrocytes are well-established to promote neuronal growth, and intracranial lipid metabolism is primarily regulated through the interaction between neurons and astrocytes. To investigate the role of astrocytes in promoting neuronal growth, we performed immunofluorescence analysis of the microtubule-associated protein 2 (MAP2) protein in neurons, as shown in Figure 3A. In the APP/PS1 conventional diet group, MAP2 expression was significantly lower than that in the control group (hippocampus: *p* = 0.0082; cortex: *p* = 0.009). While the group of APP/PS1 mice fed an OA-supplemented diet showed increased expression compared to the conventional diet group (hippocampus: *p* = 0.02; cortex: *p* = 0.002). This trend of increased MAP2 expression was consistent in both the hippocampus and cortex. Based on these findings, further experiments were conducted using astrocytes.

### 3.5 OA improved astrocyte function by modulating fatty acid β-oxidation

**Figure 5:**
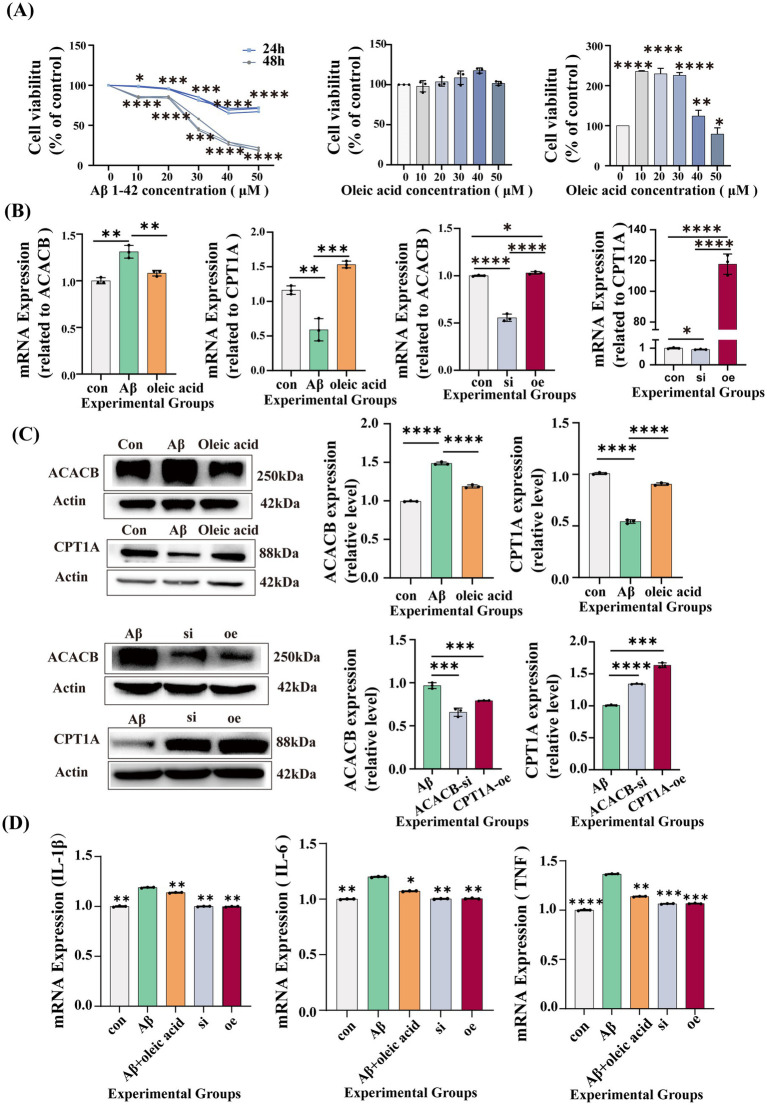
Results of cell experiments (*n* = 3). **(A)** Cell viability assays of astrocytes. Left panel 1: viability at 24 and 48 h under different concentrations of Aβ1–42. Left panel 2: viability at 48 h under different concentrations of oleic acid (OA). Left panel 3: viability at 48 h in astrocytes co-treated with Aβ1–42 and different concentrations of OA. **(B)** Gene expression analysis of ACACB and CPT1A. Left panels 1, 2: ACACB and CPT1A mRNA levels in control, Aβ1–42-treated, and OA-treated groups (ACACB: Con vs. Aβ *p* = 0.002, Aβ vs. OA *p* = 0.006; CPT1A: Con vs. Aβ *p* = 0.0045, Aβ vs. OA *p* = 0.0006). Left panels 3, 4: ACACB and CPT1A mRNA levels in control, si-ACACB, and oe-CPT1A groups. **(C)** Protein expression of ACACB and CPT1A in control, Aβ, OA, si-ACACB, and oe-CPT1A groups (ACACB: Con vs. Aβ *p* < 0.0001, Aβ vs. OA *p* < 0.0001, Aβ vs. ACACB-si *p* = 0.0008, Aβ vs. CPT1A-oe *p* = 0.0009; CPT1A: Con vs. Aβ *p* < 0.0001, Aβ vs. OA *p* < 0.0001, Aβ vs. ACACB-si *p* = 0.0008, Aβ vs. CPT1A-oe *p* = 0.0002). **(D)** mRNA expression levels of inflammatory factors in control, Aβ, OA, si-ACACB, and oe-CPT1A groups (IL-1β: Con vs. Aβ *p* = 0.0019, Aβ vs. OA *p* = 0.0018, Aβ vs. ACACB-si *p* = 0.0028, Aβ vs. CPT1A-oe *p* = 0.0019; IL-6: Con vs. Aβ *p* = 0.001, Aβ vs. OA *p* = 0.01, Aβ vs. ACACB-si *p* = 0.0018, Aβ vs. CPT1A-oe *p* = 0.002; TNF: Con vs. Aβ *p* < 0.0001, Aβ vs. OA *p* = 0.001, Aβ vs. ACACB-si *p* = 0.0005, Aβ vs. CPT1A-oe *p* = 0.0005).

We established that OA could downregulate ACACB expression in astrocytes and upregulate CPT1A expression, thereby contributing to the modulation of fatty acid metabolism. To further substantiate the effects of OA on astrocytes, an AD model of astrocytes was established using Aβ1–42. We treated astrocytes with various concentrations of Aβ1–42 for 24 and 48 h. Ultimately, we selected the concentration and time point that yielded 50% cell survival to establish the model. The final modeling conditions were set with an Aβ1–42 concentration of 30 μM for 48 h (*p* = 0.0005). To exclude the effect of OA on astrocyte survival, we treated astrocytes with different concentrations (10, 20, 30, 40, and 50 μM) of OA for 48 h. We found that various concentrations of OA did not significantly alter astrocyte survival. To determine the optimal OA treatment concentration, we treated Aβ1–42-injured astrocytes with different concentrations of OA (10, 20, 30, 40, and 50 μM) for 48 h. The 10 μM OA concentration yielded the optimal therapeutic effect (*p* < 0.0001). Ultimately, 10 μM was determined as the optimal OA treatment concentration. These results are presented in Figure 5A.

Our findings also indicated that ACACB mRNA expression was significantly higher in the Aβ1–42 intervention group compared to the control group (*p* = 0.0020). OA treatment led to a reduction in ACACB mRNA expression compared to the Aβ1–42 intervention group (*p* = 0.0060). Additionally, CPT1A mRNA expression was significantly lower in the Aβ1–42 intervention group compared to the control group (*p* = 0.0045) and was higher in the OA treatment group than in the Aβ1–42 intervention group (*p* = 0.0006). These results are shown in Figure 5B. The protein expression trends were consistent with PCR results (*p* < 0.0001), as shown in Figure 5D. We next performed ACACB knockdown and CPT1A overexpression in astrocytes (as shown in Figures 5B,C) and validated the contrasting regulatory effects using PCR and Western blotting following Aβ1–42 treatment. The protein expression of ACACB in both the si-ACACB group and the oe-CPT1A group was significantly lower than that in the Aβ1–42 group (si-ACACB vs. Aβ: *p* = 0.0008; oe-CPT1A vs. Aβ: *p* = 0.0009), as shown in Figure 5C. Moreover, CPT1A protein expression was increased in the oe-CPT1A group (*p* = 0.0002) and the si-ACACB (*p* < 0.0001) group compared to the Aβ1–42 group, as shown in Figure 5C.

**Figure 6:**
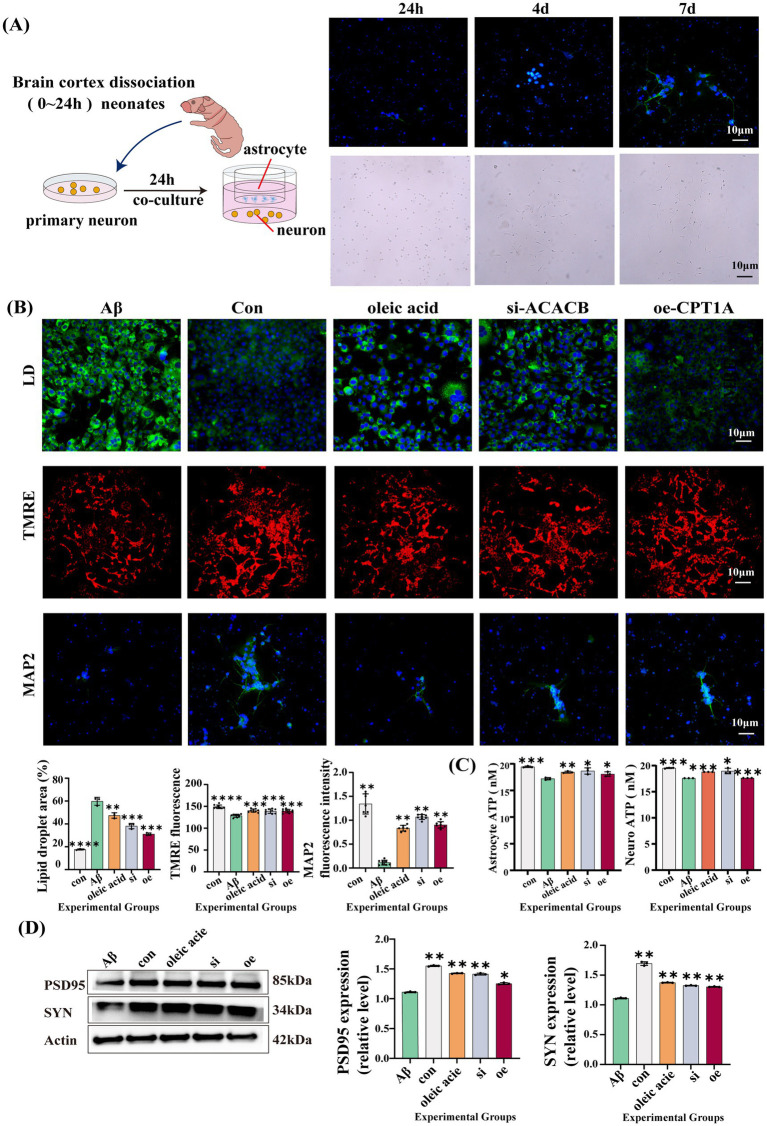
Results of astrocyte–neuron co-culture experiments. **(A)** Primary neuron isolation workflow, representative neuronal morphology at 24 h, 4 days, and 7 days, and MAP2 immunofluorescence. **(B)** Lipid droplet staining and TMRE-based mitochondrial membrane potential in five astrocyte groups (*n* = 5), and MAP2 immunofluorescence in co-cultured primary neurons (*n* = 5). (LD: Con vs. Aβ *p* < 0.0001, Aβ vs. OA *p* = 0.0018, Aβ vs. ACACB-si *p* = 0.0004, Aβ vs. CPT1A-oe *p* = 0.0005; TMEM: Con vs. Aβ *p* < 0.0001, Aβ vs. OA *p* = 0.0001, Aβ vs. ACACB-si *p* = 0.0008, Aβ vs. CPT1A-oe *p* = 0.0002; MAP2: Con vs. Aβ *p* = 0.001, Aβ vs. OA *p* = 0.0015, Aβ vs. ACACB-si *p* = 0.0058, Aβ vs. CPT1A-oe *p* = 0.0042). **(C)** ATP levels in astrocytes (left panel 1, *n* = 3) and primary neurons (left panel 2) across five groups (astrocyte: Con vs. Aβ *p* = 0.0001, Aβ vs. OA *p* = 0.0015, Aβ vs. ACACB-si *p* = 0.0158, Aβ vs. CPT1A-oe *p* = 0.0342; neuron: Con vs. Aβ *p* = 0.0009, Aβ vs. OA *p* = 0.0008, Aβ vs. ACACB-si *p* = 0.011, Aβ vs. CPT1A-oe *p* = 0.0001). **(D)** Synapse-associated protein expression (PSD95 and SYN) in co-cultured primary neurons (*n* = 3) (PSD95: Con vs. Aβ *p* = 0.0019, Aβ vs. OA *p* = 0.001, Aβ vs. ACACB-si *p* = 0.004, Aβ vs. CPT1A-oe *p* = 0.045; SYN: Con vs. Aβ *p* = 0.001, Aβ vs. OA *p* = 0.009, Aβ vs. ACACB-si *p* = 0.008, Aβ vs. CPT1A-oe *p* = 0.005).

To validate the efficacy of OA, astrocytes were assigned to five groups (1): a control group (2), an Aβ1–42 intervention group (30 μM Aβ1–42 treatment for 48 h) (3), an OA treatment group (10 μM OA treatment combined with 30 μM Aβ1–42 intervention for 48 h) (4), a si-ACACB group (ACACB gene knockdown followed by 30 μM Aβ1–42 intervention for 48 h), and (5) an oe-CPT1A group (CPT1A gene overexpression followed by 30 μM Aβ1–42 intervention for 48 h). Next, we co-cultured astrocytes with primary neurons. Based on neuronal morphology and MAP2 immunofluorescence staining, we selected a 7-day co-culture period, as shown in Figure 6A. We found that the gene expression of inflammatory factors decreased in the OA treatment group, si-ACACB group, and oe-CPT1A group, as shown in Figure 5D. Our study revealed that LD deposition was reduced in the OA treatment group, si-ACACB group, and oe-CPT1A group compared to the Aβ1–42 group. We also observed LD deposition in cells from the normal group; however, it was significantly increased in the Aβ1–42 group, as shown in Figure 6B. Furthermore, we examined changes in mitochondrial membrane potential and found that the mitochondrial membrane potential was lower in the Aβ1–42 intervention group compared to the normal group (*p* < 0.0001). While the OA treatment group showed significant improvement (*p* = 0.0001), the si-ACACB group (*p* = 0.0002) and the oe-CPT1A group (*p* = 0.0003) also showed increases. However, the potential in these intervention groups remained lower than the normal group, as illustrated in Figure 6B.

To further validate ATP production, we quantified ATP concentrations. In astrocytes, ATP production was reduced in the Aβ1–42 intervention group compared to the normal group (*p* = 0.0001). The OA treatment group improved this condition (*p* = 0.0015). Besides, ATP production was elevated in the si-ACACB (*p* = 0.0158) and oe-CPT1A (*p* = 0.0342) groups compared to the Aβ1–42 group, we similarly compared ATP concentrations of primary cells in co-culture, and ATP production was reduced in the Aβ1–42 intervention group compared to the normal group (*p* = 0.0009), which was ameliorated by the OA-treated group (*p* = 0.0008), the si-ACACB (*p* = 0.011) and oe-CPT1A (*p* = 0.0001) groups, as shown in Figure 6C.

We then conducted immunofluorescence experiments to detect the expression of the MAP2 protein in neurons. The results (Figure 6B) indicated that neuronal dendrite growth was markedly slowed in the Aβ1–42 intervention group (*p* = 0.001). In contrast, the OA treatment group (*p* = 0.0015), si-ACACB group (*p* = 0.0058), and oe-CPT1A group (*p* = 0.0042) demonstrated significantly improved dendritic growth compared to the Aβ1–42-treated group. We also confirmed the protein expression levels of PSD95 and SYN, both of which were increased in the OA treatment group, si-ACACB group, and oe-CPT1A group compared to the Aβ1–42 intervention group, as shown in Figure 6D.

**Figure 7:**
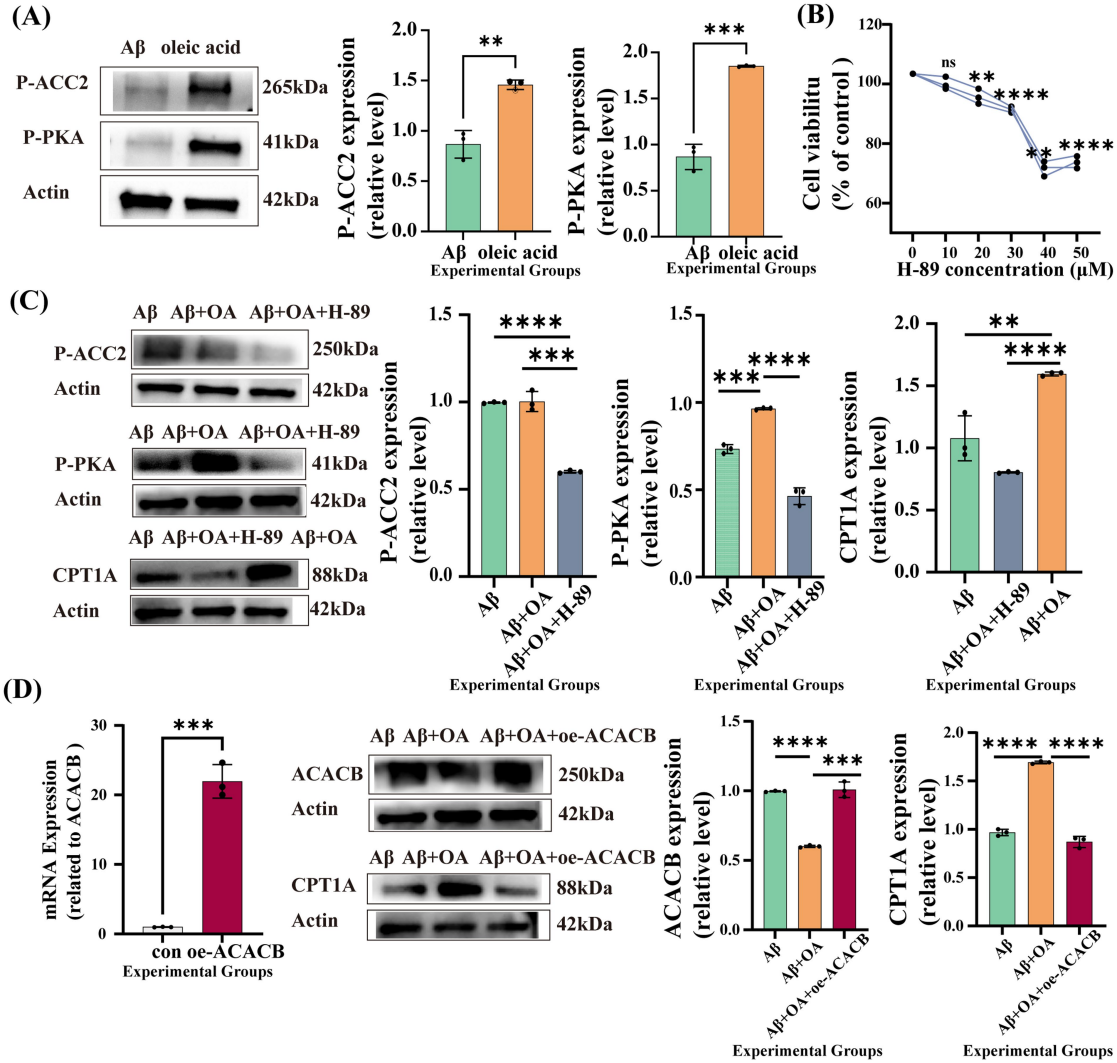
OA exerts its effects through the PKA/ACC2/CPT1A pathway. **(A)** Protein expression of P-ACC2 and P-PKA in OA-treated Aβ1–42-stimulated astrocytes (OA group) and untreated Aβ1–42-stimulated astrocytes (Aβ group) (*n* = 3; P-ACC2: Aβ vs. OA, *p* = 0.0022; P-PKA: Aβ vs. OA, *p* = 0.0002). **(B)** Cell viability after 24 h of H-89 treatment. **(C)** Protein expression after H-89 treatment (P-ACC2: Aβ vs. OA *p* < 0.0001, OA vs. Aβ + OA + H-89 *p* = 0.0003; P-PKA: Aβ vs. OA *p* = 0.0002, OA vs. Aβ + OA + H-89 *p* < 0.0001; CPT1A: Aβ vs. OA *p* = 0.0019, OA vs. Aβ + OA + H-89 *p* < 0.0001). **(D)** Left panel 1: ACACB mRNA expression after ACACB overexpression. Left panel 2: ACACB and CPT1A protein expression in the Aβ, Aβ + OA, and Aβ + OA + oe-ACACB groups (ACACB: Aβ vs. OA *p* < 0.0001, OA vs. Aβ + OA + oe-ACACB *p* = 0.0002; CPT1A: Aβ vs. OA *p* < 0.0001, OA vs. Aβ + OA + oe-ACACB *p* < 0.0001).

To explore how OA reduces ACACB expression, we validated the expression of P-PKA. We verified that due to the treatment of OA astrocytes increased the protein expression of P-PKA (*p* = 0.0002) and P-ACC2 (*p* = 0.0022) (Figure 7A), which affected the activity of ACC2. Cells were treated with H-89 following OA exposure. In preliminary experiments, H-89 alone reduced cell viability at higher concentrations; therefore, 10 μM was selected for subsequent treatment (Figure 7B). In addition, ATP production was decreased at both 24 h and 48 h in the 30 μM Aβ1–42-induced cell model (Supplementary Figure 1). To ensure effective PKA inhibition while minimizing secondary effects, a 24-h H-89 treatment duration was used. Compared with the OA-treated group, H-89-treated cells showed reduced P-PKA protein expression (*p* < 0.0001). P-ACC2 (*p* = 0.0003) and CPT1A (*p* < 0.0001) protein expression levels were also decreased, suggesting that OA regulates P-ACC2 and CPT1A, at least in part, through modulation of P-PKA (Figure 7C). An ACACB overexpression model was also established. Following ACACB overexpression and OA treatment, ACACB expression was increased (*p* = 0.0002), whereas CPT1A expression was decreased (*p* < 0.0001) (Figure 7D). ACACB overexpression attenuated the restorative effect of OA on CPT1A protein expression. Together, these findings suggest that OA regulates fatty acid metabolism in astrocytes via the PKA/ACACB/CPT1A pathway. From this, we speculated that OA could downregulate ACC2 through the PKA/ACACB pathway, thereby restoring fatty acid β-oxidation in astrocytes.

## 4 Discussion

Our research demonstrated that OA could improve cognitive function and pathological changes in APP/PS1 mice. We subsequently analyzed online databases and identified ACACB as an abnormally expressed key gene, which we verified through subsequent experiments. We identified ACACB as the target site of OA and discovered that oleic acid can influence ACC2 through the cAMP-PKA pathway, thereby impacting fatty acid metabolism in astrocytes. Overall, this is the first study to demonstrate that OA alleviates pathological changes and delays cognitive decline by regulating fatty acid metabolism in astrocytes through ACACB.

Importantly, we identified that a diet rich in MUFAs could positively impact cognitive function. Prior studies have demonstrated that unsaturated fatty acids protect against AD (Stefaniak et al., 2022). Specifically, higher levels of OA are associated with a reduced risk of progression from MCI to AD (Dakterzada et al., 2024). One investigation demonstrated that amyloid precursor protein (APP) 695 transfected Cos-7 cells supplemented with OA have reduced secreted amyloid-beta (Aβ) levels (Amtul et al., 2011). Extra virgin olive oil (the primary component of which is MUFA) can reduce Aβ deposition and tau neuropathology by enhancing autophagy and blood–brain barrier clearance mechanisms, thereby helping to prevent or even halt the progression of AD (Millman et al., 2021). Fatty acid transport across the blood–brain barrier occurs primarily through two mechanisms: passive diffusion (Mitchell and Hatch, 2011) and transporter-mediated uptake via fatty acid transport proteins. Studies have shown that the uptake of both OA and palmitic acid increases with higher expression of fatty acid transporters (Mitchell and Hatch, 2011; Raatz et al., 2018; Stahl et al., 1999). Multiple clinical studies have also identified associations between changes in the abundance of unsaturated fatty acids and AD. In these studies, both brain and plasma levels of unsaturated fatty acids were lower in AD individuals compared to cognitively normal control groups (Cunnane et al., 2012). Furthermore, studies have found that the increased plasma concentration of unsaturated fatty acids in AD patients supplemented with unsaturated fatty acids is associated with a reduction in the release of inflammatory cytokines from peripheral blood mononuclear cells (Swanson et al., 2012).

To further investigate the effects of OA on cognitive function and disease pathology, APP/PS1 mice were selected for subsequent experiments. The results confirmed that increasing OA in the diet positively affected cognitive function in mice. Our findings validate that an OA-supplemented diet improves spatial learning and working memory in APP/PS1 mice.

In brain tissue sections of 6-month-old APP/PS1 mice, we observed pathological changes, such as neuronal nuclear shrinkage, which were absent in mice fed an OA-supplemented diet. Furthermore, extensive amyloid plaque deposits were found in the brains of 6-month-old APP/PS1 mice. The deposition of amyloid plaques in the brain tissue of APP/PS1 mice fed an OA-enriched diet was significantly reduced compared to those fed a conventional diet. This suggests that dietary supplementation of OA to APP/PS1 mice could reduce plaque deposition. We hypothesized that an OA-supplemented diet could facilitate the clearance of Aβ by glial cells in brain tissue. Some studies suggest that reactive astrocytes may facilitate the processing and generation of amyloid proteins and induce inflammatory responses (Zhao et al., 2011). Both *in vitro* and *in vivo* experiments have confirmed that astrocytes can internalize and clear Aβ (Koistinaho et al., 2004; Pihlaja et al., 2008; Pihlaja et al., 2011; Nielsen et al., 2009; Nielsen et al., 2010; Deng et al., 2024). Astrocytes can express Aβ-degrading enzymes, including matrix metalloproteinases (MMPs) and insulin-degrading enzyme (IDE) (Yoshiyama et al., 2000; Palmer et al., 2009; Baig et al., 2008). Additionally, astrocytes can promote Aβ clearance through the lymphatic system (Lan et al., 2017). Our study found that 6-month-old APP/PS1 mice maintained on a diet supplemented with OA exhibited lower Glial fibrillary acidic protein (GFAP) expression than those maintained on a conventional diet. GFAP is a representative marker of astrocyte reactivity and is associated with AD pathophysiological biomarkers (Sánchez-Juan et al., 2024). We found that the inflammatory factors IL-1β, IL-6, and TNF were downregulated, therefore we speculate that OA may assist astrocytes in restoring their ability to clear Aβ.

OA can reportedly regulate fatty acid metabolism (Natali et al., 2007), and in brain tissue, astrocytes are the primary cell type of brain cells involved in fatty acid metabolism (Yin, 2023; Ioannou et al., 2019). Our study leveraged the GEO online database to analyze and identify genes associated with fatty acid metabolism. ACACB expression was identified in astrocytes using the AlzData website. The protein encoded by ACACB is ACC2, one of the key enzymes in fatty acid metabolism. It has been established that ACC2 catalyzes the conversion of acetyl-CoA to malonyl-CoA, a process that may influence the activity of CPT1 during fatty acid β-oxidation (Schreurs et al., 2010). CPT1A facilitates the transport of fatty acids for fatty acid β-oxidation (Ju et al., 2024; Lee et al., 2015). It is well-established that high expression of ACC2 inhibits the expression level of CPT1A, thereby obstructing fatty acid β-oxidation. The brain heavily depends on the fatty acid β-oxidation in astrocytes to break down fatty acids (Rudge, 2022). Experimental validation revealed that ACACB was highly expressed in the brain tissue of APP/PS1 mice, while CPT1A, a key enzymes inhibited by ACACB in fatty acid β-oxidation, was downregulated. In APP/PS1 mice fed with an OA-supplemented diet, ACACB expression was downregulated, while CPT1A expression increased in brain tissue. In an *in vitro* astrocyte model, we observed the same trend, indicating that OA could restore abnormal fatty acid β-oxidation in astrocytes under pathological conditions.

Abnormal fatty acid β-oxidation results in an increase in free fatty acids, especially free saturated fatty acids, which can lead to oxidative stress and activate inflammation (Rudge, 2022). Moreover, abnormal fatty acid β-oxidation results in LD formation in cells (den Brok et al., 2018; Currie et al., 2013). Abnormal oxidative phosphorylation in astrocytes in brain tissue also induces LD accumulation (Mi et al., 2023). LDs are organelles that contain triacylglycerols and cholesterol esters, surrounded by a layer of amphiphilic lipids and associated proteins. Under normal conditions, LD content is low in the brain. LDs store energy in the form of fatty acids, which can undergo β-oxidation to prevent the accumulation of harmful lipid intermediates and to mitigate lipotoxicity (Qi et al., 2021). Research indicates that in neurodegenerative diseases such as AD, LD levels increase (Ren et al., 2022), promoting oxidative stress and neuroinflammation (Marschallinger et al., 2020). LDs are now understood to primarily accumulate in glial cells, including astrocytes and microglia (Ralhan et al., 2021). Our previous research found that OA could restore fatty acid β-oxidation in astrocytes. We also conducted follow-up cell experiments to investigate further whether OA could improve LD deposition. Our experimental results showed that astrocytes treated with OA exhibited outcomes similar to those of astrocytes with ACACB knockdown and CPT1A overexpression, with LD accumulation reduced compared to the disease group.

Studies have demonstrated that 4-month-old 5xFAD mice exhibit defects in mitochondrial fatty acid degradation in astrocytes (Mi et al., 2023), with fatty acid β-oxidation primarily occurring in mitochondria (Yin, 2023; Ioannou et al., 2019; Fecher et al., 2019). Fatty acid oxidation in astrocytes is essential for regulating mitochondrial reactive oxygen species (ROS) production and neuronal energy adaptability (Kemp et al., 2024). Declines in mitochondrial bioenergetic function and impaired glucose metabolism are linked to brain aging, with these changes already evident in the early stages of AD. The mitochondrial metabolic profile in astrocytes is influenced more significantly by fatty acid load than by glucose levels (Mi et al., 2023) The observed data indicate that early intervention in the fatty acid degradation defect of astrocytes can restore mitochondrial function and thus delay disease progression. Our subsequent experiments further investigated the effect of OA on mitochondrial membrane potential. In cellular model experiments, we found that OA could restore decreased mitochondrial membrane potential in astrocytes derived from a disease model. Knocking down ACACB or overexpressing CPT1A could also restore mitochondrial membrane potential, suggesting that restoring fatty acid β-oxidation in astrocytes could restore mitochondrial function.

In brain tissue, fatty acid β-oxidation is a coupled process conducted by astrocytes and neurons (Chen et al., 2022). Astrocytes have been reported to synthesize lipids for neurons and absorb lipid peroxides produced by neuronal degradation (Qi et al., 2021). Disruption of this process compromises the supportive role of astrocytes for neurons (Chen et al., 2022). Allen et al. (2012) found that astrocytes promote the formation of functional excitatory synapses between neurons. We found that MAP2 expression in the hippocampus and cortex of APP/PS1 mice was significantly lower than in control mice, while MAP2 expression in APP/PS1 mice fed an OA-supplemented diet was restored. MAP2 is a cytoskeletal protein that plays a role in activity-dependent synaptic plasticity in mature hippocampal neuron networks (Kim et al., 2020). Indeed, disruption of MAP2 affects the plasticity of synaptic structure and function (DeGiosio et al., 2022), and neuronal loss is accompanied by MAP2 loss. It has been suggested that synapse loss appears to precede neuronal loss (Forner et al., 2017) and synapse loss is strongly correlated with cognitive decline in AD (Terry et al., 1991), with such changes appearing early in the disease process (Scheff et al., 2006). Astrocytes can secrete proteins and lipids to promote the formation and maturation of synapses. We discovered that primary neurons co-cultured with OA-treated astrocytes in cell co-culture experiments could restore MAP2 expression. Therefore, we propose that OA can restore fatty acid β-oxidation in astrocytes, thus restoring their supportive role in neuronal synapses.

Our study primarily focused on APP/PS1 mice aged 4 to 6 months and did not include older mice. In the future, we will extend our research to investigate the effects of an OA-supplemented diet on older mice. The cell model used in our study is the Aβ1–42-treated astrocyte cell line (C8-D1A). The modeling concentration was determined based on CCK-8 assay results, and the response of astrocytes to varying Aβ1–42 concentrations has not yet been validated. This issue will be addressed in subsequent experiments.

**Figure 8:**
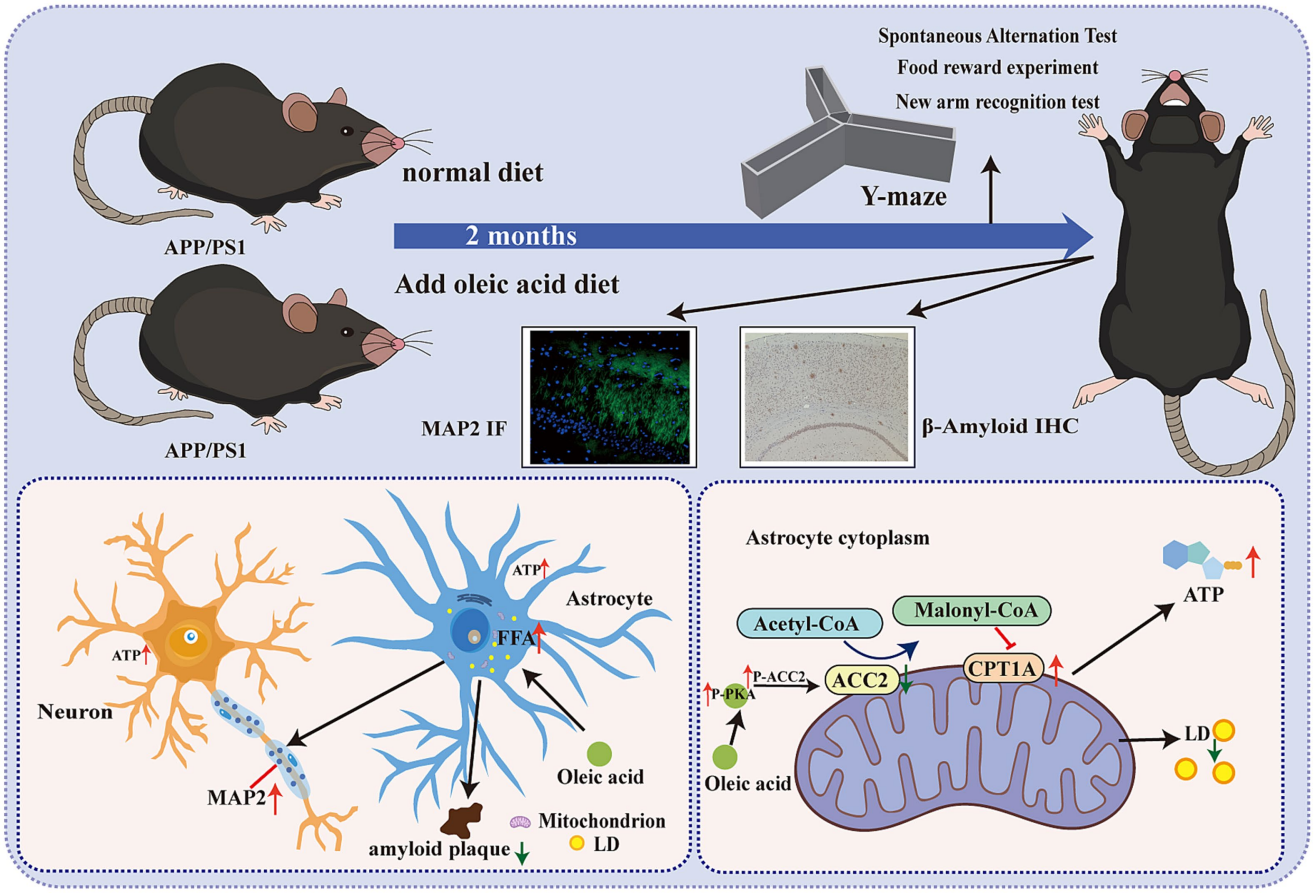
Proposed mechanism. Oleic acid increased P-ACC2 levels via upregulation of P-PKA, thereby suppressing ACC2 (ACACB) and restoring CPT1A expression. This facilitated fatty acid transport into mitochondria, enhanced astrocytic fatty acid β-oxidation, reduced lipid droplet accumulation, restored ATP production, and restored the astrocyte-mediated support of neuronal axonal growth, as well as to clear β-amyloid.

In summary, this study further confirmed that OA can diminish the inhibitory effect of ACC2 on CPT1A by suppressing ACC2 in intracranial astrocytes through the cAMP-PKA pathway, thereby restoring fatty acid β-oxidation in astrocytes. This restoration improved mitochondrial function in astrocytes, reduced abnormal LD deposition, and potentiated their ability to clear amyloid plaques while promoting and protecting neuronal growth, ultimately improving cognitive decline in AD, as shown in Figure 8. Our findings may offer insights for future dietary interventions aimed at alleviating and preventing AD.

## 5 Conclusion

Our findings suggest that OA modulates fatty acid β-oxidation in astrocytes by regulating ACACB intracranially via the PKA, thereby attenuating pathological changes and cognitive deficits in Alzheimer’s disease. Overexpression of ACACB may serve as a promising therapeutic target for the prevention and treatment of AD, and a diet supplemented with oleic acid could offer a novel dietary strategy for managing AD. Our study establishes a solid foundation for these insights. However, it has certain limitations; specifically, we did not further validate the effects of an oleic acid-supplemented diet on aged mice, which necessitates further investigation.

## Data Availability

The original contributions presented in the study are included in the article/Supplementary material, further inquiries can be directed to the corresponding author/s.

## Footnotes

1
www.cdc.gov/nchs/nhanes

2
https://www.ncbi.nlm.nih.gov/geo/

3
http://www.broadinstitute.org/msigdb

4 http://www.alzdata.org/

5
https://cn.string-db.org/
