# Supplementary material for: Oleic acid improves pathological changes in Aβ1–42-induced astrocytes and Alzheimer’s disease mouse models through PKA/ACACB/CPT1A
Source: Front Neurosci. 2026 Apr 15;20:1771310. doi: 10.3389/fnins.2026.1771310 (PMC13125043; doi:10.3389/fnins.2026.1771310)
Supplement: Supplementary file 1 [file Data_Sheet_1.PDF]

**Supplemental Table 1. Estimated Daily Caloric Intake per Mouse in Each Diet Group (n = 10, Female:Male = 1:1)**

| Group | Diet | sex | Energy density (kcal/g) | Housing (mice/cage) | Food intake (g/cage/day) | Estimated caloric load (kcal/mouse/day) |
|---|---|---|---|---|---|---|
| con | standard diet | male | 3.40 kcal/g | 5 | 17.68±2.50 | 12.02±1.70 |
| APP/PS1 | standard diet | male | 3.40 kcal/g | 5 | 18.48±2.40 | 12.57±1.63 |
| APP/PS1 +OA | diet supplemented with OA | male | 3.51 kcal/g | 5 | 17.32±2.22 | 12.16±1.56 |
| con | standard diet | female | 3.40 kcal/g | 5 | 16.27±2.17 | 11.06±1.47 |
| APP/PS1 | standard diet | female | 3.40 kcal/g | 5 | 16.05±2.20 | 10.92±1.49 |
| APP/PS1 +OA | diet supplemented with OA | female | 3.51 kcal/g | 5 | 15.64±1.64 | 10.98±1.15 |

Data are presented as estimated daily caloric intake per mouse.
Estimated caloric intake (kcal/mouse/day) was calculated as: Diet energy density (kcal/g) × [Food intake (g/cage/day) / Number of mice per cage].
Food intake was measured per cage, and values per mouse were estimated assuming equal intake among mice in the same cage (n = 5 mice per cage).

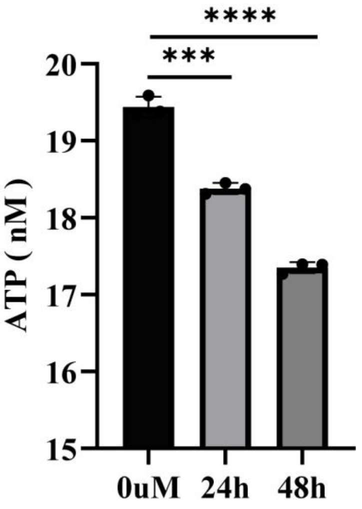


**Supplementary Figure1**

Comparison of ATP levels after 24 and 48 hours of H-89 treatment in a 30 μM Aβ1-42-induced cell model (24h vs 0h $p$ = 0.0009, 48h vs 0h $p$ < 0.0001).

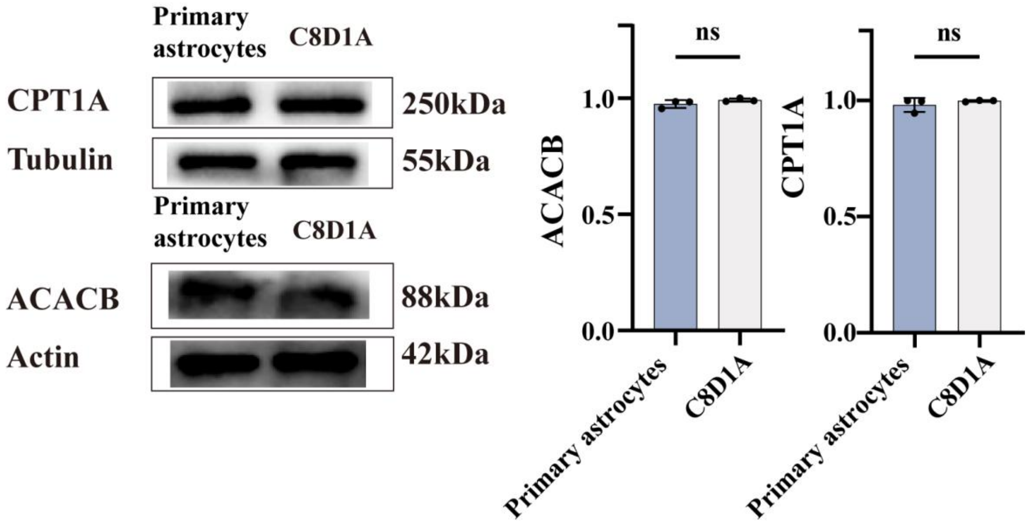


**Supplementary Figure2**

The expression of ACACB and CPT1A proteins in primary astrocytes and C8D1A cell line, $p > 0.05$.
